# A paean to the ineffable Marjory Stephenson

**DOI:** 10.1099/mic.0.001160

**Published:** 2022-03-25

**Authors:** Frank Sargent, R. Gary Sawers

**Affiliations:** ^1^​ School of Natural and Environmental Sciences, Newcastle University, Newcastle-upon-Tyne, UK; ^2^​ Institute for Biology/ Microbiology, Martin Luther University Halle-Wittenberg, Halle (Saale), Germany

## Abstract

It is now 75 years since Marjory Stephenson became the second President of the Society for General Microbiology (SGM). Around the time of her death at the end of 1948 many articles appeared extolling Marjory Stephenson’s contribution to the fields of Biochemistry and Microbiology. Not that much has been written about her since that time, which is unfortunate. Therefore, this brief review is intended as a form of redress and aims to highlight the role of this remarkable scientist in establishing the Society and in promoting Microbiology as a discipline. Notwithstanding the significance of these achievements, however, it is her overall impact on the field of ‘Chemical Microbiology’ and what she achieved through her research that are extraordinary, even by today’s standards. Marjory Stephenson recognized that in order to understand a biological system, the ‘whole’ organism must be considered and this can only be achieved by adopting an interdisciplinary approach: inorganic and organic chemistry, biochemistry, genetics, metabolism and ultimately physiology. Her scientific ethos serves today as a beacon for how scientific research should be conducted, and what we as scientists can learn about how to inspire and mentor the next generation. It is impossible to overstate Marjory Stephenson’s scientific legacy, or her overall contribution to Microbiology.

## Introduction

Cambridge Biochemistry was the brainchild of Sir Frederick Gowland Hopkins and its inception around the turn of the 20th century was accompanied by the appointment of many outstanding researchers who went on to have huge impacts on science in the UK and beyond [1, 2]. In the context of Microbiology, perhaps the most important of Hopkins’ appointments was Marjory Stephenson, whom Hopkins persuaded to come to Cambridge in 1919 to join his burgeoning new department. Hopkins’ genius was in spotting talented, inspirational and enthusiastic young scientists, nurturing them and giving them the appropriate incentive to focus on a particular biochemical topic. He then left them to develop the field themselves, acting as a mentor and ‘guiding hand’. In the early 1920s Hopkins suggested to Stephenson that she study the biochemistry of microorganisms, and that was really the beginning of ‘Chemical Microbiology’ as an independent discipline. Because Marjory Stephenson had always shown a keen interest in biochemistry (she had been a strong advocate of the Biochemical Society since just before the first World War), this stood her in good stead to tackle her new discipline with the enthusiasm and insight for which she was renowned.

During the period from 1920 until her untimely death in 1948 she, along with her dynamic group of researchers, developed to a significant degree the field of anaerobic microbiology. Clearly, other international groups were also active in this field and Stephenson was always careful to acknowledge every other researcher’s contribution. Nevertheless, it is only with hindsight and our current understanding of microbial physiology that we can really appreciate her remarkable discoveries and contributions. While her scientific brilliance is now, as it was then, unquestioned [3–5], it was also her unwavering dedication to the mentoring and promotion of young scientists that has truly become apparent in recent years. In almost every respect, Marjory Stephenson was years ahead of her time.

In this review, we shall give a brief historical retrospective about what led Marjory Stephenson to the Cambridge laboratory and how she established her research group. As the current authors are both schooled in bacterial hydrogen metabolism, some key discoveries on biohydrogen and formate made by Stephenson and her group, and which have had an immense impact on our current understanding of both chemo- and heterotrophic growth modes, will be briefly discussed. Finally, we will highlight how the Stephenson approach to mentoring of students and to science communication provides a blueprint for future generations of researchers.

## Back to Cambridge

Marjory Stephenson was born in 1885 and spent her formative years in and around Cambridge [3–5]. After obtaining her degree in Natural Science from Newnham College, she spent several years teaching before in 1911 finally getting the opportunity to do biochemical research in the laboratory of Professor R. H. Aders Plimmer at University College, London (UCL). It was here that she learnt how to analyse enzyme activity, using intestinal mucosa as a model system, and she also made in-roads into elucidating fatty acid ester biosynthesis [6]. After the 1914–18 war, when she suspended her science career and served the Red Cross in France and Greece with such distinction to be appointed Member of the Order of the British Empire [3], she returned to Cambridge, and began her long association with the Biochemistry Department, presumably receiving a strong recommendation from Plimmer to F. G. Hopkins about her experimental brilliance, her common-sense, no-nonsense approach and general enthusiasm for biochemical research.

The following, highly productive quarter century of research on bacteria at Cambridge consolidated her reputation both nationally and internationally, culminating in Marjory Stephenson’s election to the Royal Society in 1945. She and the crystallographer Dr Kathleen Lonsdale at UCL were the first women to be elected to the Society, and the esteem in which Marjory Stephenson was held by her colleagues is reflected in the number of renowned and revered scientists, including Fildes, Florey, Haldane and Keilin, to name but a few, who supported her nomination [7].

During this period at Cambridge, Marjory Stephenson was initially supported through a Beit Fellowship and then by the Medical Research Council (MRC). None of this she took for granted and her appreciation for this financial support and the scientific freedom extended by the MRC was returned by her acting as secretary of the committee on Chemical Microbiology until her death in 1948 [3–5]. Just prior to the second world war, she had developed a strong interest in nucleic acid metabolism [8], which was briefly revisited again afterwards when she worked with Jennifer M. Moyle [9]. Indeed, Stephenson is given credit for introducing Moyle to Peter D. Mitchell [10], thus forming a now legendary partnership in bioenergetics research that was to result in the development of chemiosmotic theory and a Nobel Prize.

Marjory Stephenson was a great advocate and supporter of the Biochemical Society and, as already mentioned, she was a key driver in establishing the SGM (Society for General Microbiology), which was inaugurated as a Society in 1945 [11]. While her research in Microbiology was paramount, she was also very active in teaching and apparently excelled through her infectious enthusiasm for bacteria in inspiring young scientists to join her research group. This she achieved during her supervision of advanced Biochemistry practicals, where she frequently posed challenging questions and discussed the latest research news with students [3]. This ability proved important in the recruitment of talented young students to work under her tutelage in her laboratory; she was primarily interested in advancing their knowledge and experience. Consequently, many of her former students went on to become established leaders in their respective research fields.

Marjory Stephenson’s ideas on the study of Microbiology and her dynamic biochemical approaches culminated in the publication of the textbook *Bacterial Metabolism*, which appeared in three editions, each separated by approximately a decade, with the first edition appearing in 1930 [12]. The book allowed her to place her work, and that of others, in the ‘Chemical Microbiology’ field in a broader context and would help guide and inspire students, while at the same time acting as a reference text for more experienced scientists. This textbook resulted in her receiving the international acclaim she richly deserved and helped spread the Stephenson ‘gospel’ [5].

Finally, a clear picture of how Marjory Stephenson viewed Microbiology is presented in the third and final edition of her textbook [13], and this is well worth reiterating here:

‘During the last few years, a fresh view of bacterial metabolism has been opened up. Information is now being rapidly gained on the course of the biochemical processes leading to cell synthesis; such studies are peculiar to microbiology though certainly of wider application; they owe their success to use of biological material which is prone to biochemical variation and tolerant of interference with its normal biochemical habit. This new stream of knowledge has its origin in several sources: microbial genetics, nucleic acid metabolism, adaptive enzyme formation … antibiotics … and interference with metabolism resulting from the introduction into the cell of chemical analogues of essential cell metabolites. All these are contributing to produce a picture –at present incomplete and patchy– of the biochemical machinery of growth.’

Lest we forget, this was all written before Franklin’s, and Watson and Crick’s elucidation of the structure of DNA [14, 15] and more than a decade prior to publication of Jacob and Monod’s ‘operon model’ [16]. Her opinion was that enzyme activities measured after laboratory cultivation are not necessarily the same as what is occurring in the natural environment. This rather prophetic viewpoint was not accepted by all scientists at the time; she was of course correct in her premise.

## Biohydrogen and Great Ouse mud

Although many major scientific breakthroughs were made by the Stephenson laboratory, we shall focus here on how she and her students, particularly Donald Woods and Leonard Stickland, advanced the biohydrogen research field. Marjory Stephenson realized early in her research career that in order to understand a system it is necessary to study the organism, or cell, as a complete entity. Consequently, her laboratory quickly established the use of resting cells to study metabolic processes [17]. At around the same time, the Swedish physician Torsten Thunberg had made use of the redox indicator dye methylene blue to demonstrate that redox reactions governed degradation of substrates in eukaryotic cells [cited in 5]. Based on the resting-cell technique, coupled with use of methylene blue as an artificial electron acceptor, Stephenson’s research team discovered that redox reactions were also prevalent in bacteria, providing at the same time support for Hopkins’ and Kluyver’s theory of the unity of Biochemistry [18]. It was thus becoming clear that heterotrophic ‘life’ was driven by dehydrogenation reactions [17, 19]. Marjory Stephenson preferred to work at the bench; however, she also had a keen and active group of students who helped her develop these methods over a period of nearly 20 years from the early 1920s. These students included Harry Quastel, Margaret Whetham, Leonard Stickland, Ernest Gale, John Yudkin and Donald Woods, and they helped Stephenson establish whole-cell microbial physiology as a research tool, which ultimately revealed the central role of biohydrogen in driving anaerobic microbial processes, including synthrophy [3–5].

The other key ‘player’ in establishing the field of anaerobic microbiology was a sediment sample obtained from the Great Ouse river, near Cambridge. Stephenson was inspired by an observation made nearly 50 years earlier by Felix Hoppe-Seyler, who noted that samples of river mud could transform formic acid into CO_2_ and H_2_ [reported in 20]. Being a keen ambler, she brought a mud sample with her to the laboratory after she observed that it was ‘actively fermenting’; the river had been recently contaminated by waste from a sugar-beet factory [3, 5]. Thus began her fascination with trying to understand how bacteria could grow in the absence of oxygen and, as we understand it, her foray into anaerobic microbiology. Initially, using a mixed culture derived from the Great Ouse sediment the Stephenson group showed that several substances could act as H donors and that others, such as nitrate, acted as acceptors [17, 21]. This was all based on the ability of these compounds, when added to the mixed cultures, either to reduce oxidized methylene blue to its colourless species, or to oxidize the leuco form of the dye. It was during this series of experiments that hydrogen activation by resting microbial cells was elucidated [22]. Based on their observation that hydrogen could be used as an H-donor by these cultures, Marjory Stephenson and Leonard Stickland termed the enzyme responsible for the reaction hydrogenase. The opening lines of this now famous paper (at least amongst the ‘hydrogenase community’) give insight into the quintessence of Marjory Stephenson’s scientific approach:

‘Biochemists are now accustomed to regard the transfer of hydrogen as an essential step in biological oxidations; such a view involves the conception of some enzymic mechanism for rendering active or unstable the molecule from which the hydrogen is transferred. No such enzyme acting on molecular hydrogen has so far been described, though several inorganic catalysts are known which function in this way. It is nevertheless almost certain that such an enzyme exists, as organisms producing molecular hydrogen presumably have such a catalyst.’

The laboratory then isolated pure anaerobically growing cultures, initially by cultivation in rich medium. These pure cultures were then tested in chemically defined medium, ultimately allowing the resting cells derived from them to be analysed with different H donors and acceptors. Having identified these enzymic activities, she and her group were then able to delve deeper into the mechanisms of these processes by making use of her previously acquired biochemical knowledge. This was an important step in the advent of microbial physiology. Using these now classical approaches, this led her group to discover in a coliform bacterium (probably a species of the genus *Escherichia *) the H_2_-evolving formate hydrogenlyase (FHL) reaction [20] and substrate-dependent (‘adaptive’) enzyme induction [20, 23], an idea Stephenson cited as being first propounded by H. Karström [24]. The approach also allowed the first quantitative descriptions of H_2_-dependent sulphate reduction [25], H_2_-dependent methane production (methanogensis) [26] and the implication of interspecies hydrogen transfer during heterotrophic growth [20, 23, 27]. In his Marjory Stephenson prize lecture, Rudolf Thauer acknowledged Stephenson’s discovery as ‘the beginning of the modern era for the study of methanogenesis’ [28] and this also marked the first description of the isolation in pure culture of a methanogenic archaeon, although the evidence that archaea are a separate kingdom from bacteria and eukarya would take a further 50 years to become established [29].

The other key approach adopted by the Stephenson group was the use of manometry to allow quantitative assessment of biochemical reactions. The importance of this approach to their work was evident from the statement that ‘… whatever may be the function of the hydrogen-hydrogenase system in the cell it is a remarkably useful tool in the hands of the bacterial chemist, for by its use bacterial reductions can be studied rapidly and quantitatively by manometric methods and the products of reduction obtained unmixed with the products of oxidation.’ An example of the simple and quantitative use of the manometer was Stephenson and Stickland’s demonstration [25] that during sulphate reduction by *Desulfovibrio desulfuricans,* 4 moles of H_2_ were necessary to reduce 1 mole of sulphate, according to:

$$H_{2}SO_{4}+4\;H_{2}\to H_{2}S+4\;H_{2}O$$

They also demonstrated in the study that thiosulphate and sulphite could be reduced to hydrogen sulphide using H_2_ [25], thus identifying sulphite as an intermediate in the sulphate reduction pathway and thiosulphate as an alternative electron acceptor.

The identification of the H_2_-evolving FHL reaction in *Escherichia coli* also proved to be a milestone in more ways than one. This was one of the most lucid examples of substrate-dependent enzyme induction, or ‘adaptive’ (as opposed to ‘constitutive’) enzyme synthesis, as Stephenson termed it [20, 23], and substantiated the earlier demonstration by Stickland [30] that the FHL reaction required the participation of two enzyme components, namely a formate dehydrogenase and a hydrogenase. Although the authors remained equivocal about whether it represented a single enzyme complex (as we now know it to be) or comprised a two-enzyme ‘pathway’, it was clear that addition of formate to resting cells (i.e*.* no cell division) was required to induce the activity. In the authors’ own words, ‘washed suspensions rapidly dehydrogenated formic acid in the presence of methylene blue, but did not carry out the reaction resulting in the liberation of hydrogen gas until they had been in contact with the formate solution for some 24 h.’ [20]. They showed that growth in the presence of oxygen prevented H_2_ evolution, they predicted that there was a distinct enzyme (we know this to be pyruvate formate-lyase [31]) that generated formate, they demonstrated that H_2_ production was better at low pH, and they showed that 1 mole of formate was disporportionated into 1 mole each of H_2_ and CO_2_. Although not referred to as such, this was the first prescient description of the formate regulon [32]. In that same paper, Stephenson and Stickland also provided the first evidence for a separate nitrate-dependent formate dehydrogenase [20]. It was also later shown by Woods, while still in Stephenson’s group, that the FHL reaction was reversible [33]. Stephenson’s laboratory later substantiated the theory of substrate-dependent induction of enzyme synthesis for galactose degradation in both *Saccharomyces cerevisiae* [34] and *E. coli* [35].

The comparatively few papers (more were not required) resulting from her laboratory’s work were concisely written, packed full of quantitative information about the subject and included insightful interpretations of their data. For example, she correctly proposed, based on the interpretation of kinetic data, that *E. coli* had more than one hydrogenase [22].

Whenever another group or researcher had made earlier observations pertinent to the topic under study, Stephenson always described and accredited these in the first few sentences of the manuscript. In their paper describing the hydrogenlyase reaction [20], the first sentence of the paper begins, ‘The earliest work dealing with the bacterial decomposition of formic acid was that of Hoppe-Seyler [1876], who dealt with its decomposition into carbon dioxide and hydrogen by mixed cultures obtained from mud …’, and the Introduction to their paper on hydrogen-dependent sulphate reduction [25] states that ‘It was originally shown by Beijerinck [1895] that the hydrogen sulphide produced in mud arises anaerobically by the bacterial reduction of sulfates’. This generous, and rightful, recognition and attribution of the significance of colleagues’ work was a hallmark of Marjory Stephenson and her laboratory.

## A view of the biohydrogen field 75 years on

An assessment of all the advances made in all of the research fields to which Marjory Stephenson made a significant contribution during her career would result in several hefty tomes. Consequently, we will only make very brief mention here of some of the major advances in bacterial and archaeal hydrogen metabolism brought about in the last few decades and which follow up her key initial breakthroughs.

The period between the end of the 1970s and the beginning of the 1980s was highly productive in terms of the isolation and characterization of hydrogenases. Indeed, it was confirmed for a number of bacterial and archaeal species that they synthesized more than one hydrogenase enzyme, as predicted by Stephenson and Stickland for *Escherichia* and *Desulfovibrio* species. These multiple hydrogenase enzymes are often employed in recapturing H_2_ released during fermentative metabolism, either by the same bacterium or within the immediate community. This was elegantly shown for sulphate-reducers [36], for *E. coli* [37, 38], for hydrogenotrophic methanogens [39] and for *Clostridium pasteurianum* [40].

A dependence on the transition metal nickel for methanogensis revealed hydrogenase, along with methyl-CoM reductase [39], to have a nickel cofactor that was initially predicted, and later demonstrated, to have a direct role in H_2_ activation [41]. During the mid-1980s nickel was also identified as a component of the hydrogenases of *E. coli* [38], revealing that this metal was prevalent in the cofactor of hydrogenases from other species. However, analysis of clostridial hydrogenase II revealed that it contained no nickel and only iron. These two classes of enzyme differ phylogenetically [42] and to this was added a third broad class of iron-only hydrogenases found so far exclusively in certain methanogenic archaea [43]. The hydrogenases of clostridia and single-celled eukarya such as *Chlamydomonas reinhardtii* have in common that they lack [NiFe]-hydrogenases.

The first crystal structure of a [NiFe]-hydrogenase was determined for the enzyme from the sulphate-reducer *Desulfovibrio gigas* [44] and this study revealed that the [NiFe]-cofactor included unprecedented diatomic ligands (CO and CN^−^) attached to the iron ion. This has been confirmed meanwhile for all [NiFe]-hydrogenases [41] and is a common feature of these enzymes [45]. The structural analysis of [FeFe]-hydrogenases [46] has revealed that, despite their phylogenetic distinction from the [NiFe]-hydrogenases, they nevertheless share structural and mechanistic similarities ([FeFe]-hydrogenase also requires diatomic ligands in its active site cofactor), a lucid example of convergent evolution. The elucidation of the mechanisms of diatomic ligand biosynthesis has revealed new biochemical reactions that probably indicate an ancient origin for these enzymes and for their biosynthesis.

The genetic analysis of the FHL complex also delivered unexpected new ‘biology’, including the discovery of selenocysteine as the 21^st^ proteinogenic amino acid [47, 48], as well as the routes of nickel uptake and incorporation into bacterial enzymes, including urease [49]. Analysis of the sequences of the genes encoding FHL revealed that the complex shared a common evolutionary ancestor with the mitochondrial NADH dehydrogenase (Complex I), cementing the enzyme’s important place in the evolution of cellular energy metabolism [50]. Meanwhile, advances in molecular biology and anaerobic purification techniques have resulted in the isolation of the FHL complex from *E. coli* [51], thus allaying all equivocation about it being a ‘single’ enzyme. Recent research has also confirmed and extended the initial observation made by Woods [33] that the FHL reaction is reversible, opening up new possibilities for biotechnological production of the energy-rich intermediate formate as an alternative fuel [52].

Finally, in the last few years it has been clearly established that hydrogenases afford many bacterial species the capability of utilizing environmental levels of hydrogen as an energy source [53]. This verifies another observation made by Stephenson and Stickland in their landmark hydrogenase papers in the early 1930s [22]. Hydrogen metabolism also plays a central role in the animal gut microbiome, and hydrogenases are important for both colonization by commensal bacteria and in infection by bacterial pathogens [54, 55]. Marjory Stephenson would be astounded at these advances.

## Marjory Stephenson the innovator, mentor and teacher

What can be gleaned from Marjory Stephenson’s scientific papers reveals that she was very precise, showed considerable common-sense, clearly thought about the laboratory’s data very carefully, and was succinct, almost laconic; she did not ‘mince her words’. Much information on Marjory Stephenson the educator is provided by S. R. Elsden, N. K. Pirie, M. Robertson and D. D. Woods who knew her well and who had the unenviable task of writing her obituary [3–5]. What we learn from these ‘mini-reviews’ of her life and her achievements is that she was tireless in her support of Biochemistry and Microbiology, initially in Britain, but through her enthusiasm, her renown and particularly her textbook *Bacterial Metabolism* this spread internationally. She was also extremely loyal, particularly to her students, but most especially to Frederick Hopkins.

Woods wrote in his obituary of Marjory Stephenson that ‘Her genius was for experiment rather than theory …’ [3]. This statement was meant to convey that she did not indulge in great speculation about the possible meaning of the data presented in her papers. She avoided writing a ‘Discussion’ section and instead preferred to let the data speak for itself; she was the personification of an empiricist and this was reflected in the guidance of her students.

Although she was keen to impart her knowledge of Biochemistry to students, according to Woods [3] she was apparently not a fan of formal lectures, which is somewhat ironic in light of the institution of the ‘Marjory Stephenson Prize Lecture’ in her honour. Instead, she preferred the bench-interaction with students during practical courses, where she could discuss and explain her views on Microbiology. According to Elsden and Pirie [5], Stephenson was a great advocate of Pasteur’s view that ‘a new idea was of more importance than old knowledge’. Consequently, she took no heed of criticism that her methods of teaching neglected taking into account taxonomy, or that she lacked a basic training in bacteriology. She rightly recognized very quickly that in order to understand how a bacterium, or any organism for that matter, grows and survives, it is necessary ultimately to study it in its natural habitat. She realized early that there are often greater metabolic differences within a single culture during different phases of growth than between different species. Indeed, she wrote in 1937 that ‘… during the growth of cultures the medium itself is constantly changing, some constituents disappearing and others appearing, it is easy to see that the enzyme activity of growing and even non-growing cultures in the ferment vats must be changing continuously …’ [56]. Stephenson thus understood the significance of metabolic adaptation, and the value of careful observation and data analysis. Stephenson’s approach to science is also what inspired Jacob and Monod [14]. Nevertheless, Marjory Stephenson promulgated the idea of first following a biological process or reaction in mixed culture, then in pure culture, then in crude extracts and finally with the purified enzyme: ‘No one level was, by itself, adequate; and for an understanding of bacteria as they are found in Nature, research must occur at all levels’ [5].

It was this far-sighted learning experience and the knowledge gained from its implementation that Marjory Stephenson tried to impart to future generations of scientists. As recorded by Woods [3], her adage was ‘infection not instruction is the secret of education’.

The number of scientists who studied in the Stephenson laboratory and then developed their own careers is testament to her exacting method of mentoring her students: Set them a challenge, discuss the approaches that could be used to answer the question to be addressed, and give them a gentle push to start them off. The key was that she gave her students enough independence, even to the extent of letting them publish without her name on the paper, to maximize the learning experience, but she was always on hand, if necessary, ‘to extricate them in the end’ [3].

## Champion of the Society for General Microbiology

All the while that Marjory Stephenson was teaching and performing innovative benchwork, she was continually championing Chemical Microbiology. She became secretary of the MRC committee on Chemical Microbiology in 1929 and continued in this role until her death. She was also a member of the Biochemical Society for 35 years and was a member of its committee from 1928 to 1932. Marjory Stephenson pushed hard for the formation of Chemical Microbiology as a twin society with the Biochemical Society. She was part of an organizing committee of approximately 30 microbiologists, which held many preliminary meetings, beginning in 1943 and was instrumental in naming the new Society [11]. The inaugural meeting of the new Society for General Microbiology was convened in February 1945 at the London School of Hygiene and Tropical Medicine and 250 votes in favour of forming the Society were cast, which was quite a respectable turnout for a first meeting of the SGM, and this included a letter of support from the then President of the Royal Society Sir Henry Dale [11]. The organizing committee was unanimously elected, as was the first President, Sir Alexander Fleming. Marjory Stephenson declined a request by committee members to become the first President of the SGM, presumably on account of her ill-health, but was persuaded to become its second President in 1947. This was also the year that the *Journal of General Microbiology* (now *Microbiology*) was founded.

Marjory Stephenson is an iconic figure both in her support for women in science and in helping establish an outreach programme aimed at explaining the importance of bacteria and biochemistry to the general public. This took the form of a series of public radio broadcasts in 1930. The success of these broadcasts is reflected in a quotation made by Ernest Gale and Paul Fildes in their obituary notice marking the death of Donald Woods [57], where they quote him as stating ‘I became interested in this subject (chemical microbiology) at 8.30 p.m. on Friday 9 May 1930. At that time the late Dr. Marjory Stephenson gave a broadcast talk in a series entitled “Biochemistry: what it is and what it does”. Her particular topic was “How microbes live or some aspects of bacterial physiology”. This short talk made a deep impression upon me; I was 18 at the time, which is no doubt an impressionable age …’.

Marjory Stephenson also maintained strong links with Newnham College throughout her academic life and became a member of its Governing Body in 1931 and of its council from 1944. Moreover, she found time to support the Association of Scientific Workers where she acted as Vice-President in the 1940s [5]. These facets of Marjory Stephenson’s life reveal much about her character. She was a vigorous defender of the role of women in science and society. In this regard Frederick Hopkins’ insight and recognition of Marjory Stephenson’s abilities should also be recognized, because he was the one who had the courage to give her the opportunity to become effectively one of the very first women in a senior managerial role in science. She was ideally suited to this role and she had the strength of character to follow her scientific instincts and not kow-tow to pressure exerted by senior MRC members to work on ‘more medically relevant topics’. Her will prevailed and Microbiology would not have made the advances it did, as soon as it did, without her. Reverential tones are often used when referring to some of the renowned scientists, such as Haldane, Krebs and Szent-Györgyi, who passed through Hopkins’ Cambridge Biochemistry Institute. Marjory Stephenson’s name should also be uttered with the same reverence.

**Fig. 1.**
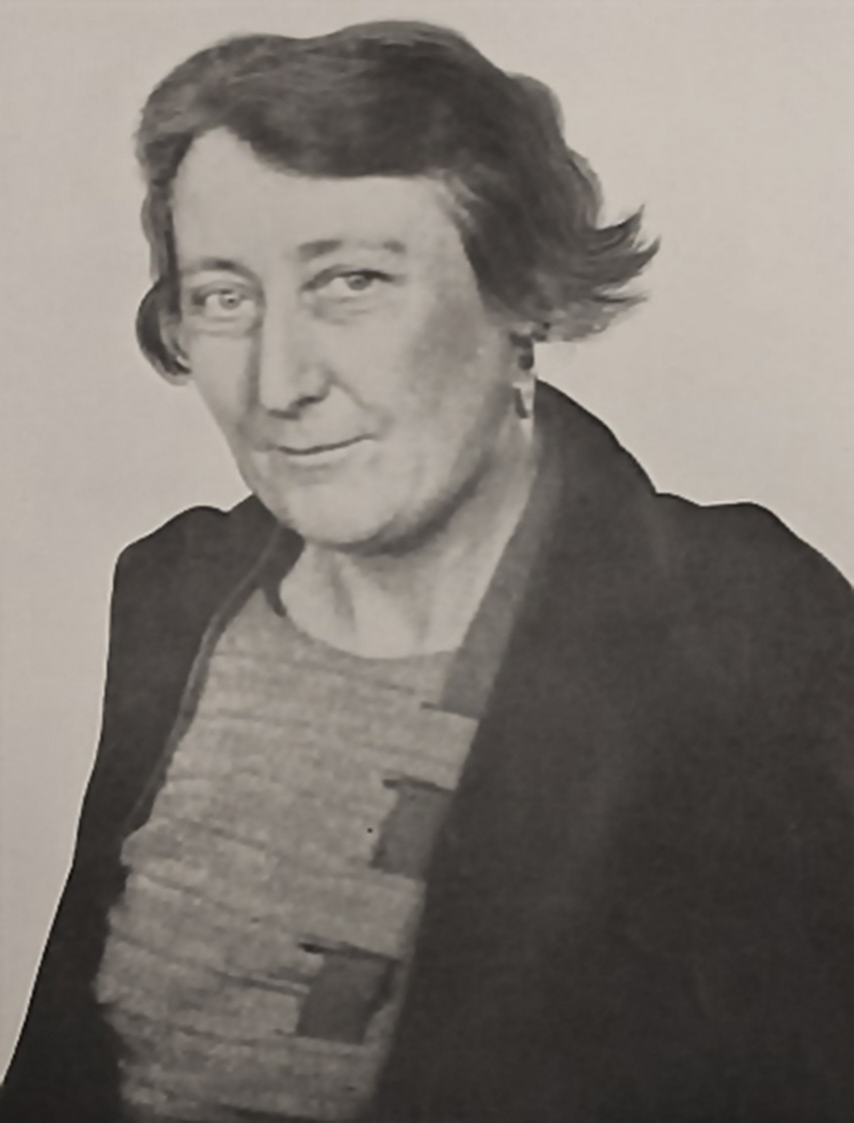
Marjory Stephenson. Image credit: Wellcome Collection, the Society for General Microbiology archives; adapted from photo album 'The History of the Biochemistry Department - Volume 1', University of Cambridge, collected for Sir Frederick Gowland Hopkins, early 1930s
